# [Corrigendum] Chemopreventive effects of PBI‑Se, a selenium‑containing analog of PBIT, on AOM‑induced aberrant crypt foci in F344 rats

**DOI:** 10.3892/or.2025.8877

**Published:** 2025-02-17

**Authors:** Naveena B. Janakiram, Altaf Mohammed, Durgadevi Ravillah, Chang In Choi, Yuting Zhang, Dhimant Desai, Shantu Amin, Chinthalapally V. Rao

Oncol Rep 30: 952–960, 2013; DOI: 10.3892/or.2013.2483

Following the publication of the above article, an interested reader drew to the authors' attention that the RT-PCR data panels shown in Fig. 3C, showing the effects of PBIT and PBI-Se treatment on endogenous IL-8 mRNA expression levels in CaCo_2_ cells, contained issues. Specifically, on p. 956, a pair of the β-actin bands in the bottom panel of Fig. 3C were strikingly similar to those featured in the upper panel. Upon examining their original data, the authors realized that the bottom β-actin panel in Fig. 3C had inadvertently been assembled incorrectly. Moreover, a typo was also identified in the Fig. 3C legend.

The revised version of Fig. 3, showing the correct data for the β-actin bands in the bottom panel in Fig. 3C, and now accurately displaying the correct images for the IL-8 mRNA and β-actin expression experiments, is shown on the next page. Additionally, the typo in the Fig. 3C legend has been corrected to read as follows: ‘PBI-Se (1–4 µM) and PBIT (30–60 µM) treatment reduced endogenous IL-8 mRNA expression in CaCo2 cells’. Note that the revisions made to this figure do not affect the overall results or the conclusions reported in the paper. The authors are grateful to the Editor of *Oncology Reports* for granting them the opportunity to publish this corrigendum, and all the authors agree with its publication; furthermore, they apologize to the readership of the journal for any inconvenience caused.

## Figures and Tables

**Figure 3.**
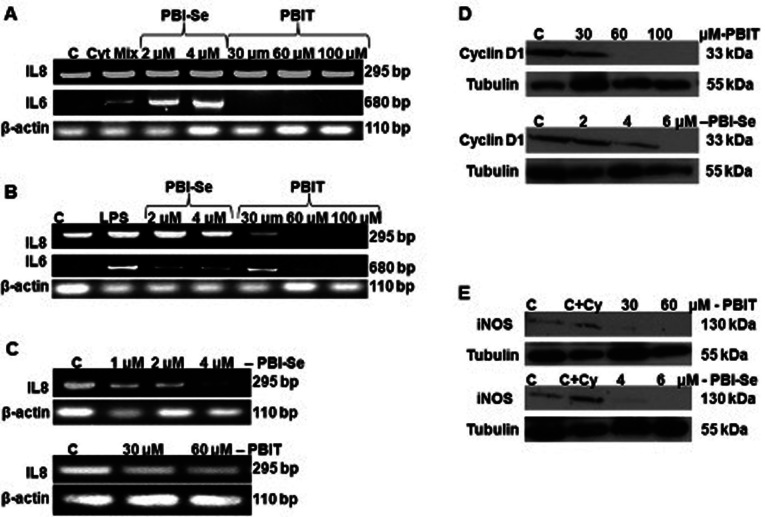
Effect of PBI-Se (2–4 μM) and PBIT (30–100 μM) on IL-8 and IL-6 mRNA expression induced by (A) a cytokine mixture or (B) by LPS. PBIT suppressed IL-8 and IL-6 mRNA expression induced in the human colon cancer CaCo2 cell line by the cytokine mixture and suppressed IL-6 mRNA production induced by LPS. PBI-Se also suppressed LPS-induced IL-6 mRNA expression, but enhanced IL-6 production in response to the cytokine mixture. (C) PBI-Se (1–4 μM) and PBIT (30–100 μM) treatment reduced endogenous IL-8 mRNA expression in CaCo2 cells. (D) CaCo2 cells were treated with increasing concentrations of PBI-Se or PBIT for 24 h and cell lysates were immunoblotted with antibodies against cyclin D1 and α-tubulin. Both drugs were effective in decreasing the cyclin D1 protein expression. (E) CaCo2 cells were treated with PBI-Se (at 4 and 6 μM) and PBIT (at 0, 30, 60 and 100 μM) for 24 h. Cell lysates were immunoblotted using antibodies against iNOS and α-tubulin. Both the drugs were effective in suppressing iNOS protein expression..

